# Speechreading in Deaf Adults with Cochlear Implants: Evidence for Perceptual Compensation

**DOI:** 10.3389/fpsyg.2017.00106

**Published:** 2017-02-07

**Authors:** Hannah Pimperton, Amelia Ralph-Lewis, Mairéad MacSweeney

**Affiliations:** ^1^Institute of Cognitive Neuroscience, University College LondonLondon, UK; ^2^Deafness, Cognition and Language Centre, University College LondonLondon, UK

**Keywords:** speechreading, deaf, cochlear implants, compensation, lipreading

## Abstract

Previous research has provided evidence for a speechreading advantage in congenitally deaf adults compared to hearing adults. A ‘perceptual compensation’ account of this finding proposes that prolonged early onset deafness leads to a greater reliance on visual, as opposed to auditory, information when perceiving speech which in turn results in superior visual speech perception skills in deaf adults. In the current study we tested whether previous demonstrations of a speechreading advantage for profoundly congenitally deaf adults with hearing aids, or no amplificiation, were also apparent in adults with the same deafness profile but who have experienced greater access to the auditory elements of speech via a cochlear implant (CI). We also tested the prediction that, in line with the perceptual compensation account, receiving a CI at a later age is associated with superior speechreading skills due to later implanted individuals having experienced greater dependence on visual speech information. We designed a speechreading task in which participants viewed silent videos of 123 single words spoken by a model and were required to indicate which word they thought had been said via a free text response. We compared congenitally deaf adults who had received CIs in childhood or adolescence (*N* = 15) with a comparison group of hearing adults (*N* = 15) matched on age and education level. The adults with CI showed significantly better scores on the speechreading task than the hearing comparison group. Furthermore, within the group of adults with CI, there was a significant positive correlation between age at implantation and speechreading performance; earlier implantation was associated with lower speechreading scores. These results are both consistent with the hypothesis of perceptual compensation in the domain of speech perception, indicating that more prolonged dependence on visual speech information in speech perception may lead to improvements in the perception of visual speech. In addition our study provides metrics of the ‘speechreadability’ of 123 words produced in British English: one derived from hearing adults (*N* = 61) and one from deaf adults with CI (*N* = 15). Evidence for the validity of these ‘speechreadability’ metrics come from correlations with visual lexical competition data.

## Introduction

The perceptual compensation hypothesis refers to the idea that sensory deprivation within one sensory modality will stimulate compensatory perceptual improvement in another sensory modality (Ronnberg, 1995). Individuals who are deaf have compromised, and sometimes minimal, access to the sounds that make up a spoken language via the auditory modality. However, when a speaker produces speech, visual, as well as auditory, information about speech sounds is available to the observer. This raises the possibility that deaf individuals may show spontaneous perceptual compensation in the domain of speech perception, with their greater reliance on the visual elements of speech in everyday life resulting in superior speech perception skills in the visual-only modality. If this perceptual compensation hypothesis is correct, we would predict that deaf individuals would show superior speechreading (visual-only speech perception) skills to hearing individuals at a group level. However, evidence regarding whether there exists a speechreading advantage for deaf individuals has been mixed.

A body of work by Ronnberg et al. (1983) with individuals who had acquired hearing loss in adulthood found no evidence for superior speechreading skills in these adults compared with hearing adults (Lyxell and Ronnberg, 1989, 1991). The results of these studies led Ronnberg to conclude that “daily dependence on lipreading in a variety of social situations does not seem to suffice as a trigger for the development of speech-reading skill” (Ronnberg, 1995). Tye-Murray et al. (2007) examined speechreading of phonemes, words and sentences in older adults with mild-moderate hearing loss acquired in adulthood and compared their performance to older adults without hearing loss. In a visual-only condition they found no significant advantages for the adults with hearing loss on phonemes or sentences, but did find that they displayed a significant advantage over the adults without hearing loss in terms of their visual recognition of single words.

In contrast with the findings on adults with acquired hearing loss, studies with groups of adults who have congenital or early onset deafness have been more consistent in demonstrating significant speechreading advantages compared to hearing adults. Bernstein et al. (2000) examined the ability of adults with normal hearing (*N* = 96) and with severe to profound early onset (94% experienced onset ≤ 4 years) deafness (*N* = 72) to speechread consonant-vowel nonsense syllables, words and sentences. The adults with early onset deafness showed enhanced speechreading ability relative to the hearing adults on all three types of speechreading stimuli, indicative of superior visual phonetic perception in the deaf adults. Auer and Bernstein (2007) replicated this finding of a significant speechreading advantage for adults with early onset deafness. They compared the performance of a large group (*N* = 112) of adults with early deafness (onset < 4 years) with that of a group of hearing adults (*N* = 220) on a sentence-level speechreading task. They found significant advantages for the deaf adults who identified 43.55% of the target words correctly compared to only 18.57% for the hearing group. They concluded that “the need to rely on visual speech throughout life and particularly for the acquisition of spoken language by individuals with early onset hearing loss, can lead to enhanced speechreading ability.” Similar results were reported by Mohammed et al. (2006) using the Test of Adult Speechreading, a speechreading test that assesses speechreading skill at different levels of linguistic complexity and that was designed specifically to give deaf and hearing individuals an equal chance to demonstrate their speechreading skill by not requiring spoken or written responses. They found significant speechreading advantages for a group of 29 profoundly deaf adults (age of onset < 5 years) over a comparison group of 29 hearing adults. In a study of Brazilian Portuguese-speaking adults, Oliveira et al. (2014) found similar advantages for deaf adults over hearing adults in terms of their performance on a range of speechreading tasks, and consistent advantages for those deaf adults with prelingual onset as compared to those with post-lingual onset.

A range of skills are likely to underpin this speechreading advantage in those born deaf. In particular it is clear that individual differences in cognitive skills play an important role in speechreading skill (for review see Rönnberg et al., 2013). For example, Rönnberg et al. (1999) reported a case study of speechreading ‘expert’ – MM. They report that MM’s speechreading skill was associated with high cognitive skills, such as phonological skills and working memory capacity.

Better visual speech understanding in individuals with congenital or early onset deafness, compared to hearing individuals, but not in those with later onset of deafness is consistent with work on perceptual compensation in blind individuals. Gougoux et al. (2004) found superior pitch discrimination skills in early blind adults (blinded < 2 years old) compared to sighted adults but no evidence of these enhancements to listening skills in late blind adults (blinded > 5 years old). They also reported a significant negative correlation between age of blindness onset and pitch discrimination performance, with those who were blind from an earlier age showing superior performance on the pitch discrimination task, and argued that “cerebral plasticity is more efficient at early developmental stages” (Gougoux et al., 2004). Subsequent studies have controlled for the influence of musical experience by including sighted controls closely matched on musical experience and have still provided consistent evidence regarding the enhancement to pitch discrimination associated with earlier onset of blindness (Wan et al., 2010).

### Speechreading in Cochlear Implant Users

In the majority of the studies reviewed above that demonstrated a speechreading advantage in adults born severely to profoundly deaf, the participants either used hearing aids or no hearing device (Bernstein et al., 2000; Mohammed et al., 2006; Auer and Bernstein, 2007). Thus, these individuals would have had minimal access to the auditory speech signal meaning that their dependence on visual speech to access spoken language would have been high. Aparicio et al. (2012) have demonstrated that this visual speech signal can be enhanced by the use of cued speech (CS) which requires the user to pay more attention to the lips. They tested deaf CS and non-CS users and hearing participants on a sentence to picture speechreading test. Deaf participants who were native CS users were better speechreaders than deaf participants who were non-CS users. Furthermore, the two groups of deaf participants were better speechreaders than the hearing participants. This study demonstrates that different language and communication experiences in deaf individuals can lead to differences in speechreading skill.

Another way to increase the clarity of the speech signal to a deaf person is of course to increase access to the auditory input. Over the last two decades increasing numbers of profoundly deaf children and adults have received cochlear implants (CI); devices which convert acoustic stimuli into electrical signals and directly stimulate the auditory nerve to provide deaf individuals with access to sound (American Speech-Language-Hearing Association, 2004). For individuals who are congenitally deaf but are implanted in early childhood, or for those who receive a CI following an acquired hearing loss, it is often the case that the CI gives them sufficient access to speech sounds for them to be able to recognize speech in auditory-only conditions, although there is considerable variability in speech perception outcomes even within these populations (American Speech-Language-Hearing Association, 2004). This raises the question of whether the superior access to auditory speech that deaf CI users experience impacts on their ability to perceive visual speech. It is possible that a lesser degree of dependence on the visual perceptual elements of speech for understanding spoken language means that the group-level deaf speechreading advantage may not be evident for CI users.

However, it is important to recognize that a CI uses a maximum of 22 electrodes to replace the function of around 16,000 hair cells and as a consequence conveys highly impoverished information about speech sounds compared to a normally functioning human cochlea (Giraud et al., 2001; Nittrouer et al., 2012). The reduced spectral information conveyed by the CI is particularly problematic in terms of its impact on auditory speech perception in the presence of background noise (Srinivasan et al., 2013). This suggests that despite the increased access to auditory speech that a CI can bring, CI users might continue to make greater use of visual speech information than hearing individuals and thus may display a speechreading advantage.

The study by Oliveira et al. (2014) described above included some deaf participants with CIs but they were grouped together with participants without CIs, so it was not possible to differentiate whether the individuals with CIs displayed a group advantage relative to the hearing controls in terms of their speechreading skills. A small number of studies have reported data comparing speechreading skills of groups of deaf individuals who have received a CI with hearing individuals. Rouger et al. (2007) assessed speechreading performance in a group of post-lingually deafened adults using a task in which participants had to identify and repeat bisyllabic words presented in a visual only format. The participants completed the assessment both prior to receipt of a CI, immediately after switch on and in the years subsequent to implantation. They found that the deaf adults (*N* = 97) showed significantly higher speechreading performance than a comparison group of hearing adults (*N* = 163) when they were assessed prior to cochlear implantation. This advantage maintained in the months and years following cochlear implantation despite these deaf adults substantially increasing their auditory-only word recognition abilities. Additionally, Rouger et al. (2007) reported on a small sample (*N* = 8) of participants who had experienced sudden onset deafness less than a year before they received a CI and who still showed significantly superior speechreading performance compared to the hearing participants prior to, and following, cochlear implantation. They argued on the basis of this that “a high level of speechreading ability can be acquired rapidly during a period of auditory deprivation,” a position in stark contrast to that of Ronnberg (1995).

As part of a study looking at audiovisual spoken word training, Bernstein et al. (2014) reported scores on a sentence-level lipreading task (Auer and Bernstein, 2007) for a sample of pre- or perilingually profoundly deaf adults (*N* = 28) with CIs, the majority of whom received their CI in adulthood (>19 years), and a sample of hearing adults (*N* = 43). As was the case in the original Auer and Bernstein (2007) study, Bernstein, Eberhardt and Auer found a significant advantage for the deaf group over the hearing group in terms of their ability to identify words from sentences presented in a visual-only format; the average percentage words correct for the CI group was 39.4%, compared to 8.1% for the hearing group.

Huyse et al. (2013) compared the performance of congenitally, profoundly deaf children with CIs (*N* = 31; *M* age = 10 years, *SD* = 0.47) with that of hearing children (*N* = 31; *M* age = 10 years, *SD* = 0.5) on a task that required them to identify vowel-consonant-vowel nonsense syllables. They reported no significant differences between the groups in terms of their identification performance when the syllables were presented in a visual-only format, indicating no speechreading advantage for these deaf children with CIs over their hearing peers. This finding is consistent with the results from a study by Kyle et al. (2013) which compared the speechreading skills of a more audiologically diverse group of deaf children (severely to profoundly deaf, and using CIs, hearing aids or no device) with those of hearing children using a speechreading test that assessed visual speech recognition at multiple levels of linguistic complexity (words, sentences, and short stories). They found that the deaf and hearing children performed very similarly on this test; there were no significant differences between the groups on any of the three subtests. It is possible that increased experience of and attention to visual speech over a period of years is necessary for the development of superior visual speech perception skills observed in adults. Alternatively, it may be the case that the language skills of deaf children limit their performance on speechreading tasks, making it harder for them to demonstrate an advantage in their visual speech perception skills than it is for deaf adults with more experience of the spoken language the tests are conducted in.

In this study we therefore focused on deaf adults and aimed to test whether the group-level speechreading advantage demonstrated by Rouger et al. (2007) in post-lingually deafened adults who received their CIs in adulthood, and by Bernstein et al. (2014) in pre- and perilingually deafened adults who received their CIs in adulthood, could be replicated in a group of congenitally deaf adults who received their CIs in childhood or adolescence. We predicted that although these adults may on average have experienced greater access to auditory speech sounds than adults with equivalent levels of deafness without CIs they would still have experienced, and be continuing to experience, a much greater dependence on visual speech information than hearing individuals and hence would show evidence of perceptual compensation and demonstrate group-level advantages in their speechreading skills compared to hearing adults.

As mentioned above, auditory speech perception outcomes following implantation are highly variable and are impacted by a number of different variables. For post-lingually deafened adults, factors identified as predictors of auditory speech perception following implantation include duration of pre-implant deafness and residual hearing pre-implant (Blamey et al., 1992; van Dijk et al., 1999). For prelingually deaf children, age at implantation, residual hearing pre-implant, non-verbal ability, and exposure to an oral education have been identified as factors related to variation in speech perception outcomes following implantation (O’Donoghue et al., 2000; Svirsky et al., 2004; Geers et al., 2008). Under the framework of perceptual compensation, it would be predicted that individual variability in auditory speech perception with a CI may relate to individual variability in visual speech perception, with those individuals getting the least auditory speech access via their CI relying the most on visual speech on a day to day basis and hence showing the greatest enhancements in their visual speech perception skills.

In the present study we focus on one variable that has been consistently associated with variability in auditory speech perception outcomes following CI; age at implantation. Age at implantation effects on speech perception outcomes have been discussed in the context of sensitive periods for the development of the central auditory system. Sharma et al. (2002) have argued that the first 3.5 years of life is a period of maximal plasticity of the central auditory system. They found evidence from electrophysiological recordings of cortical auditory evoked potentials (CAEPs) that, for congenitally deaf children, implantation after 3.5 years is associated with an increased risk of developing atypical CAEPs following implantation, with these atypical CAEPs particularly likely with implantation after the age of 7 years (Sharma et al., 2002). These findings suggest that receipt of an implant earlier in life may be associated with better auditory speech perception as a consequence of increased plasticity of the central auditory system. Additionally, earlier recipients will also have experienced an increased number of years accessing auditory speech via the CI than later recipients. Taken together these factors may contribute to earlier implanted individuals showing a reduced reliance on, and therefore less well-developed, visual speech perception skills than later implanted individuals.

One study has addressed this question of whether there is evidence of a relationship between age at implantation and speechreading ability and has done so in children with CIs: Bergeson et al. (2005) compared the visual, auditory, and audiovisual speech perception performance of a group of earlier implanted (≤4 years 5 months) children with those of a group of later implanted (>4 years 5 months) children both before and in the years following implantation. They found that overall the earlier implanted children showed better speech perception performance than the later implanted children when sentences were presented in auditory-only conditions, but that this advantage was reversed when the sentences were presented in visual-only conditions with the later-implanted group showing superior performance in that context. These findings in children with CIs are consistent with the perceptual compensation hypothesis in indicating that a more protracted period of deafness, with onset in early childhood, may be associated with superior visual speech perception skills. In the present study we sought to test this hypothesis in adults with CIs who received their implant at highly variable ages (2–19 years). The majority of children who are eligible for a CI today are now receiving one before the age of 3 years (Raine, 2013) meaning that opportunities to address questions about the implication of variability in age of implantation that spans beyond the sensitive period for the development of the central auditory system are increasingly limited; thus this sample presents a unique opportunity.

To summarize, in the present study we tested the following hypotheses:

(1) Profoundly, congenitally deaf adults with CIs would show a significant advantage in their single word speechreading skills compared to a matched group of hearing adults.
(1) Within the group of adults with CIs, age at implantation would relate to speechreading skill, with earlier implanted adults showing less good speechreading skills.

## Materials and Methods

### Participants

Sixty one native English-speaking hearing participants provided data for this study. All reported normal hearing and normal or corrected-to-normal vision. The participants were either undergraduate students participating for course credit or volunteers from the wider community who had responded to adverts to take part in the study. All provided informed consent prior to participation in the study. An additional 13 participants consented to participate in the study but were excluded from the current dataset as a result of not completing all items in the task.

Fifteen congenitally deaf participants with CIs participated in this study. Age at implantation ranged from 2 to 19 years (*M* = 8.27, *SD* = 5.05). All reported normal or corrected-to-normal vision and profound deafness.

To faciliate comparisons between the speechreading performance of CI and hearing participants, each of the 15 CI participants was individually matched to a hearing participant from the larger hearing sample (*N* = 61) on the basis of age and education level, with these 15 matched hearing participants forming the hearing comparison group (HCG). There were no significant differences in the distribution of ages between the CI (median = 23, range = 22–26) and HCG (median = 23, range = 20–31) groups (*U* = 122, *Z* = 0.41, *p* = 0.71, *r* = 0.07). The groups also did not differ significantly in terms of the distribution of highest education level achieved [χ^2^(2, *N* = 30) = 0.16, *p* = 0.92, φ_c_ = 0.07].

### Materials and Procedure

One hundred and twenty three words were selected as the target words for this experiment (see **Supplementary Table S1** for full list). All words were either concrete nouns or colors. Information on the visual speech lexical competition experienced by the speechread target words was sourced from the Phi-Lex database (Strand, 2014). The measure used was a continuous measure of visual lexical competition (ConV). This measure reflected the overall competition in the reference lexicon for the target word based on the similarity of the response distributions of its constituent phonemes (from a forced choice visual only phoneme identification task) to those of phonemes in every other word of the same pattern type in the lexicon. For further details of how this measure was derived, see Strand (2014). This variable was available for 86 out of the 123 words in the study.

**FIGURE 1:**
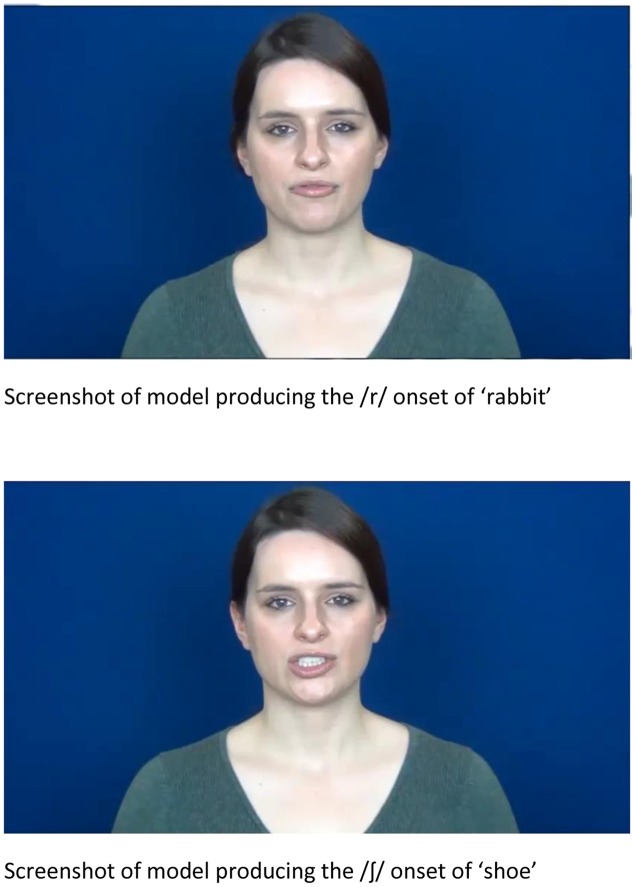
**Screenshot of visual speech model who produced all stimuli**.

A video of each word being spoken by a female model was made using a Sony Handycam (HDR-CX115). The word was spoken aloud at a normal conversational volume during the recording and the videos were subsequently edited to mute the volume such that the participants saw a natural production of the word but without any sound. The same model produced each word. The model maintained a neutral facial expression in the production of every word and the camera distance, lighting and background conditions were consistent for each word (see **Figure 1**).

Four different randomized orders of the 123 videos were produced and participants were randomly assigned to complete one of the four orders. The videos were presented using Opinio, a web-based survey tool, and participants completed the task via the internet on their personal computers. Participants were instructed that they would see silent videos of a model saying a single word and that they could only view each video once. They were required to click to play each video and then write the word they thought they had seen in a free text response box before moving onto the next video.

When scoring the responses as correct or not relative to the target, participants were given one point for an item if the response either directly matched the target or if they had produced a homophone of the target (e.g., had written ‘I’ for the target ‘eye’). This meant they could score a maximum of 123 on the task.

Prior to completing the speechreading task the participants provided demographic information via the web-based tool. Additional audiological information was collected from the participants with a CI via a paper-based response form prior to their completion of the speechreading task.

## Results

### Overall Speechreading Performance of Hearing Participants

The mean number of words identified correctly by the 61 hearing participants was 22.38 (*SD* = 9.94; range = 2–48) out of 123. This was equivalent to a mean proportion correct of 0.18 (*SD* = 0.08; range = 0.02–0.39). The mean number of words identified correctly by the hearing participants was significantly above the floor of 0 [*t*(60) = 17.56, *p* < 0.001, *d* = 2.25] and significantly below the ceiling of 123 [*t*(60) = -79.08, *p* < 0.001, *d* = -10.12].

### Comparison of Speechreading Performance for the CI Participants and the Hearing Control Group (HCG)

**FIGURE 2:**
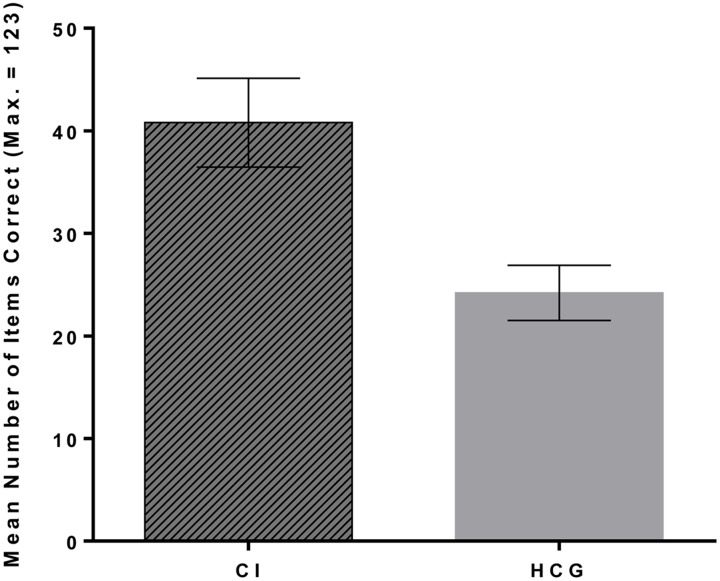
**Mean number of items correctly identified by the Cochlear Implant participants (CI) and the hearing control group (HCG).** *N* = 15 in each group.

The mean number of words identified correctly by the CI participants was 40.80 (*SD* = 16.81; range = 9–62) and by the matched HCG was 24.20 (*SD* = 10.40; range = 4–46) (see **Figure 2**). This was equivalent to a mean proportion correct of 0.33 (*SD* = 0.14) for the CI group and 0.20 (*SD* = 0.08) for the HCG. Levene’s test indicated unequal variances between the two groups. Therefore the unequal variance Welch *t*-test was used. This showed that deaf CI users scored significantly higher than the hearing control group on the speechreading task, *t*(23.35) = 3.25, *p* = 0.003, *d* = 1.19. The mean number of items correct for the matched HCG used for this group comparison (*N* = 15; *M* = 24.20, *SD* = 10.40) did not differ significantly from that of the remaining hearing participants (*N* = 46; *M* = 21.78, *SD* = 9.83), [*t*(59) = 0.82, *p* = 0.42, *d* = 0.24], suggesting that the level of performance of these matched hearing participants was representative of the performance level of the wider hearing sample.

### Relationship between Speechreading Performance and Age at Implantation

**FIGURE 3:**
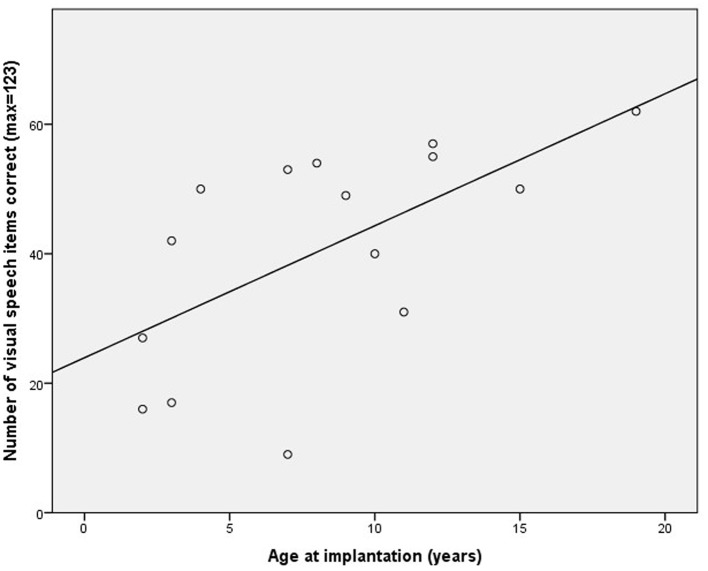
**Correlation between age of cochlear implantation in the deaf participants and number of visual speech words correctly identified**.

Within the CI group there was a significant positive correlation between age at implantation and score on the speechreading task (*r* = 0.61, *p* = 0.02, 95% CI = 0.29–0.84), with those participants who received their CIs at a later age showing higher speechreading scores than the earlier implanted participants (see **Figure 3**). A regression analysis predicting speechreading task score with age and education level entered at Step 1 and age at implantation at Step 2 indicated that age at implantation accounted for significant unique variance in speechreading performance, accounting for 30% of the variance over and above the 11% accounted for by age and education level (**Table 1**).

**Table 1:** Results of regression analyses predicting speechreading score with age and education level entered at Step 1 and age at implantation at Step 2.

	*R*^2^	*R*^2^ change	*F* change (*p*)	Final standardized β *(p)*
*Step 1*	0.11	0.11	0.77 (0.49)	
Age				
Education level^+^				
*Step 2*	0.41	0.30	5.51 (0.04)	0.001 (0.99)
Age				0.196 (0.50)
Education level^+^				0.56 (0.04)
Age at implantation				


*^+^Dichotomised into < or ≥ undergraduate degree. Age at implantation accounted for unique variance in the speechreading score.*

### Individual Item Accuracy and Relationship with Word Properties

For the 61 hearing participants, the proportion correct for individual items ranged from 0.77 (for ‘rabbit’) to 0.00 (for ‘bull’; ‘duck’; ‘fan’; ‘jacket’; ‘mat’; ‘milk’; ‘shorts’; ‘skirt’; ‘trousers’; ‘van’; ‘wall’; ‘wheel’). **Supplementary Table S1** presents data on the proportion correct responses for each of the 123 items individually for the hearing sample (**Supplementary Table S1**). These data provide a metric of ‘speechreadability’ of the 123 words when produced in British English.

There was a high degree of concordance between the hearing participants and the deaf participants in terms of the relative success rates on the individual items [*r*(121) = 0.79, *p* < 0.001, BCA bootstrapped 95% CI = 0.70–0.85]. For the 15 deaf participants with CIs, proportion correct for individual items ranged from 0.87 (for ‘elephant’; ‘fish’; ‘lorry’; ‘orange’; ‘phone’; ‘rabbit’) through to 0.00 (for ‘ball’; ‘bee’; ‘bull’; ‘duck’; ‘fan’; ‘hand’; ‘hen’; ‘ladder’; ‘mat’; ‘milk’; ‘pan’; ‘peg’; ‘red’; ‘ring’; ‘shorts’; ‘wall’; ‘wheel’; ‘wing’). **Supplementary Table S2** presents data on the proportion correct responses to each of the 123 items for the participants with CIs.

Relationships between the proportion of correct responses to an item (PropCorr) and word properties of that item were examined for the 86 words that had continuous visual lexical competition data (ConV) available for them. The distribution of the PropCorr and ConV variables showed deviations from normality so bias corrected and accelerated bootstrapped 95% confidence intervals (1000 bootstrap samples) are presented for the correlation coefficients. The number of correct responses for the items showed a significant negative correlation with ConV for both the hearing participants [*r*(84) = -0.39, *p* < 0.001, BCA bootstrapped 95% CI = -0.55 to -0.18] and the CI participants [*r* (84) = -0.50, *p* < 0.001, BCA bootstrapped 95% CI = -0.64 to -0.34] indicating that words that had fewer visual (visemic) lexical competitors were more successfully identified by both groups of participants.

## Discussion

The first aim of this study was to compare the single word speechreading ability of hearing adults and deaf adults with CIs in order to test the hypothesis that prelingually deaf adults who received an implant in childhood or adolescence would show a speechreading advantage. We found that the mean score on the speechreading task was significantly higher for the adults with CIs than for the matched comparison group of hearing adults. The performance of the matched HCG did not differ significantly from that shown by the larger sample of hearing participants who were not selected for the HCG, suggesting HCG performance was representative of the broader hearing sample. This finding of a speechreading advantage for the deaf adults with CIs in this study is consistent with the findings of Rouger et al. (2007) and Bernstein et al. (2014) who found evidence of a deaf speechreading advantage for adults with later age at implantation or later age at onset of deafness. These consistent findings in adults with CIs suggest that even with the greater access to the auditory elements of speech that a CI provides, these adults are still substantially more dependent on visual speech than hearing adults and consequently have developed compensatory superiority in their ability to use visual speech information to understand spoken language. It would be interesting in future studies to contrast this group with deaf adults without CI to further understand the extent of this compensation.

In terms of the locus of this compensation, the finding in this study of an advantage for the adults with CIs on a single word speechreading task in which there was no sentential context suggests that the deaf speechreading advantage is not exclusively driven by an enhanced ability to use sentence-level contextual information to facilitate identification of individual words. This is not to say that the use of top down sentence-level processing to aid speechreading is not something that deaf adults develop the capacity to use to effectively support speechreading in real word contexts, but rather that they also have enhanced skills in domains that support the type of context-free lipreading performance assessed in this task. This suggests that they may be better at visually perceiving individual phonemes. Alternatively, they may be better either at perceiving rapid sequences of phonemes or at using coarticulatory information in phoneme sequences to disambiguate visual phonetic information (e.g., influences of voiced vs. voiceless consonants on preceding vowel length; although the phonemes /t/ and /d/ are visually perceptually identical in isolation they have a differential effect on the articulation of the middle vowel when in a word final position; contrast ‘beat’ and ‘bead.’) Future studies should aim to test this by comparing the performance of hearing adults and deaf adults on a speechreading task in which they have to speechread both individual phonemes (e.g., ‘/f/’) and also non-words which use the phonotactics of the ambient spoken language (e.g., ‘mip,’ ‘niddy’).

The second aim of this study was to test the hypothesis that, within the CI group, later implantation would be associated with superior speechreading skills. We found a significant positive correlation between age at implantation and speechreading performance, indicating a speechreading advantage for those implanted at later ages. This result is consistent with the hypothesis that a greater duration or degree of dependence on visual information for speech perception leads to improved visual speech perception skills and therefore with the idea of perceptual compensation in the domain of speech perception. The finding of superior visual speech perception skills in later implanted adults is also consistent with the findings of Bergeson et al. (2005), who reported superior visual-only speech perception in later-implanted children (in contrast to superior auditory speech perception skills in earlier-implanted children). However, it is important to acknowledge this was a preliminary study in adults with CIs with the small sample size meaning that the confidence intervals for this correlation are wide and therefore replication of this result in a larger sample would be of value. A further limitation of this study was the lack of detailed audiological information available for the CI participants. We had no objective audiological measures for the CI users. Thus it was not possible to determine whether the earlier-implanted participants did have superior auditory speech perception through their implant than the later-implanted participants, as was the case in the Bergeson et al. (2005) study with children, and whether this related to their visual speech perception skills.

It was also the case that we did not have extensive information regarding the language skills of the CI participants. It is possible that variability in underlying spoken language knowledge within the CI group may have influenced their performance on the speechreading task and hence the relationship between age at implantation and speechreading score. However, this would be dependent on the later implanted participants showing superior spoken language skills to the earlier implanted participants, a situation which is the reverse of that typically observed. Additionally, the language demands in this speechreading task were relatively low. Participants identified single words, all of which were early acquired concrete nouns, meaning that the contributions of existing language knowledge to task performance are likely to have been minimized.

The speechreading task used in this study was completed remotely via the internet. Unfortunately this meant that we were unable to collect measures of participant’s other cognitive skills known to be important to individual differences in speechreading skill, such as phonological skills and working memory capacity (see Rönnberg et al., 1999, 2013). Future studies should attempt to include these measures. Remote data collection also meant that we were unable to monitor participants’ attention while they undertook the task. We reasoned that if participants showed item-level response patterns that were consistent with the predictions of models of spoken word recognition in the visual modality, this would support the validity of this task as a measure of speechreading. Activation-competition models of spoken word recognition in the auditory modality (e.g., the Neighborhood Activation Model; Luce and Pisoni, 1998) have posited that hearing a spoken word elicits simultaneous activation of multiple words in the mental lexicon that are perceptually similar to the target word and compete with the target word for recognition, the result being that words with more perceptually similar ‘neighbors’ are recognized with less accuracy than words that have fewer such neighbors in auditory word recognition paradigms (Dirks et al., 2001). Analogously, research examining spoken word recognition in the visual modality has shown equivalent effects of visual lexical competition experienced by the target word on recognition accuracy suggesting that activation-competition models of word recognition extend beyond the auditory modality (Feld and Sommers, 2011; Strand and Sommers, 2011). For both the CI and hearing groups in the present study, visual lexical competition showed a significant negative relationship with recognition accuracy for the words; those words with greater visual lexical competition were correctly recognized less often than those with lower visual lexical competition. This suggests that the single word speechreading task was measuring a consistent construct across the two participant groups, and supports the validity of this task as a measure of visual speech perception.

## Conclusion

The results of this small scale study provided two strands of evidence that are consistent with perceptual compensation in the domain of speech perception. First, prelingually deaf adults with CIs showed a significant advantage in terms of their performance on a visual-only speechreading task compared to a comparison group of hearing adults matched on age and education. Second, age at implantation within the CI group showed a significant positive relationship with performance on the speechreading task; participants who received their implants later in life showed superior visual-only speech perception skills. We argued that in both cases these patterns of findings resulted from increased dependence on visual speech information leading to compensatory improvements in perception of speech via the visual modality.

## Ethics Statement

This research was approved by the University College London Research Ethics Committee. Online informed consent was obtained from each participant.

## Author Contributions

HP and MM designed the study. AR-L collected the data. HP analysed the data. HP and MM wrote the paper.

## Conflict of Interest Statement

The authors declare that the research was conducted in the absence of any commercial or financial relationships that could be construed as a potential conflict of interest.
