# Supplementary material for: Speechreading in Deaf Adults with Cochlear Implants: Evidence for Perceptual Compensation
Source: Front Psychol. 2017 Feb 7;8:106. doi: 10.3389/fpsyg.2017.00106 (PMC5294775; doi:10.3389/fpsyg.2017.00106)
Supplement: Supplementary file 1 [file Table_1.docx]

**Pimperton, Ralph-Lewis, MacSweeney**

**Supplementary materials**

Table S1. Proportion correct responses for each item for the hearing participants (N = 61)

| Word | Proportion correct |
| --- | --- |
| \| **rabbit** \| \| --- \| \| **fish** \| \| **orange** \| \| **blue** \| \| **mouth** \| \| **apple** \| \| **brush** \| \| **phone** \| \| **lorry** \| \| **elephant** \| \| **spoon** \| \| **cow** \| \| **Ice cream** \| \| **cake** \| \| **tree** \| \| **rope** \| \| **eye** \| \| **knife** \| \| **light** \| \| **ship** \| \| **yellow** \| \| **bath** \| \| **car** \| \| **boat** \| \| **fork** \| \| **carrot** \| \| **pen** \| \| **telephone** \| \| **boot** \| \| **coat** \| \| **house** \| \| **road** \| \| **shoe** \| \| **aeroplane** \| \| **banana** \| \| **cloud** \| \| **green** \| \| **pea** \| \| **balloon** \| \| **train** \| \| **chair** \| \| **helicopter** \| \| **jumper** \| \| **toast** \| \| **arm** \| \| **ball** \| \| **sheep** \| \| **bush** \| \| **bird** \| \| **glasses** \| \| **leaf** \| \| **shop** \| \| **umbrella** \| \| **dress** \| \| **ear** \| \| **hat** \| \| **pig** \| \| **bone** \| \| **cave** \| \| **clock** \| \| **knee** \| \| **rose** \| \| **bun** \| \| **cars** \| \| **clown** \| \| **hammer** \| \| **leg** \| \| **ring** \| \| **snowman** \| \| **soap** \| \| **bee** \| \| **cage** \| \| **ghost** \| \| **goat** \| \| **ham** \| \| **jam** \| \| **key** \| \| **kite** \| \| **map** \| \| **shirt** \| \| **sock** \| \| **star** \| \| **tea** \| \| **tractor** \| \| **chip** \| \| **finger** \| \| **hand** \| \| **lion** \| \| **saw** \| \| **tap** \| \| **cows** \| \| **dog** \| \| **door** \| \| **king** \| \| **mop** \| \| **red** \| \| **snake** \| \| **bat** \| \| **bell** \| \| **moon** \| \| **peg** \| \| **pink** \| \| **spider** \| \| **wing** \| \| **zip** \| \| **hen** \| \| **ladder** \| \| **lamb** \| \| **monkey** \| \| **pan** \| \| **scissors** \| \| **bull** \| \| **duck** \| \| **fan** \| \| **jacket** \| \| **mat** \| \| **milk** \| \| **shorts** \| \| **skirt** \| \| **trousers** \| \| **van** \| \| **wall** \| \| **wheel** \| | \| 0.77 \| \| --- \| \| 0.74 \| \| 0.66 \| \| 0.64 \| \| 0.64 \| \| 0.56 \| \| 0.54 \| \| 0.54 \| \| 0.52 \| \| 0.51 \| \| 0.51 \| \| 0.49 \| \| 0.49 \| \| 0.48 \| \| 0.46 \| \| 0.43 \| \| 0.39 \| \| 0.39 \| \| 0.38 \| \| 0.38 \| \| 0.38 \| \| 0.36 \| \| 0.34 \| \| 0.33 \| \| 0.33 \| \| 0.31 \| \| 0.31 \| \| 0.31 \| \| 0.28 \| \| 0.25 \| \| 0.25 \| \| 0.25 \| \| 0.25 \| \| 0.23 \| \| 0.23 \| \| 0.23 \| \| 0.23 \| \| 0.23 \| \| 0.21 \| \| 0.21 \| \| 0.20 \| \| 0.20 \| \| 0.20 \| \| 0.20 \| \| 0.18 \| \| 0.18 \| \| 0.18 \| \| 0.16 \| \| 0.15 \| \| 0.15 \| \| 0.15 \| \| 0.15 \| \| 0.15 \| \| 0.13 \| \| 0.13 \| \| 0.13 \| \| 0.13 \| \| 0.11 \| \| 0.11 \| \| 0.11 \| \| 0.11 \| \| 0.11 \| \| 0.10 \| \| 0.10 \| \| 0.10 \| \| 0.10 \| \| 0.10 \| \| 0.10 \| \| 0.10 \| \| 0.10 \| \| 0.08 \| \| 0.08 \| \| 0.08 \| \| 0.08 \| \| 0.08 \| \| 0.08 \| \| 0.08 \| \| 0.08 \| \| 0.08 \| \| 0.08 \| \| 0.08 \| \| 0.08 \| \| 0.08 \| \| 0.08 \| \| 0.07 \| \| 0.07 \| \| 0.07 \| \| 0.07 \| \| 0.07 \| \| 0.07 \| \| 0.05 \| \| 0.05 \| \| 0.05 \| \| 0.05 \| \| 0.05 \| \| 0.05 \| \| 0.05 \| \| 0.03 \| \| 0.03 \| \| 0.03 \| \| 0.03 \| \| 0.03 \| \| 0.03 \| \| 0.03 \| \| 0.03 \| \| 0.02 \| \| 0.02 \| \| 0.02 \| \| 0.02 \| \| 0.02 \| \| 0.02 \| \| 0.00 \| \| 0.00 \| \| 0.00 \| \| 0.00 \| \| 0.00 \| \| 0.00 \| \| 0.00 \| \| 0.00 \| \| 0.00 \| \| 0.00 \| \| 0.00 \| \| 0.00 \| |
